# Evaluation of diastolic function by three-dimensional volume tracking of the mitral annulus with cardiovascular magnetic resonance: comparison with tissue Doppler imaging

**DOI:** 10.1186/s12968-014-0071-3

**Published:** 2014-09-20

**Authors:** Vincent Wu, Janice Y Chyou, Sohae Chung, Sharath Bhagavatula, Leon Axel

**Affiliations:** Department of Radiology, Bernard and Irene Schwartz Center for Biomedical Imaging, New York University School of Medicine, 660 First Avenue, Room 411, New York, NY 10016 USA; Department of Medicine, Leon H. Charney Division of Cardiology, New York University School of Medicine, New York, NY USA

**Keywords:** Diastolic function, Mitral annulus, Cardiovascular magnetic resonance, Echocardiography, Feature-tracking

## Abstract

**Background:**

Measurement of mitral annulus (MA) dynamics is an important component of the evaluation of left ventricular (LV) diastolic function; MA velocities are commonly measured using tissue Doppler imaging (TDI). This study aimed to examine the clinical potential of a semi-automated cardiovascular magnetic resonance (CMR) technique for quantifying global LV diastolic function, using 3D volume tracking of the MA with conventional cine-CMR images.

**Methods:**

124 consecutive patients with normal ejection fraction underwent both clinically indicated transthoracic echocardiography (TTE) and CMR within 2 months. Interpolated 3D reconstruction of the MA over time was performed with semi-automated atrioventricular junction (AVJ) tracking in long-axis cine-CMR images, producing an MA sweep volume over the cardiac cycle. CMR-based diastolic function was evaluated, using the following parameters: peak volume sweep rates in early diastole (PSR_E_) and atrial systole (PSR_A_), PSR_E_/PSR_A_ ratio, deceleration time of sweep volume (DT_SV_), and 50% diastolic sweep volume recovery time (DSVRT_50_); these were compared with TTE diastolic measurements.

**Results:**

Patients with TTE-based diastolic dysfunction (n = 62) showed significantly different normalized MA sweep volume profiles compared to those with TTE-based normal diastolic function (n = 62), including a lower PSR_E_ (5.25 ± 1.38 s^−1^ vs. 7.72 ± 1.7 s^−1^), a higher PSR_A_ (6.56 ± 1.99 s^−1^ vs. 4.67 ± 1.38 s^−1^), a lower PSR_E_/PSR_A_ ratio (0.9 ± 0.44 vs. 1.82 ± 0.69), a longer DT_SV_ (144 ± 55 ms vs. 96 ± 37 ms), and a longer DSVRT_50_ (25.0 ± 11.0% vs. 15.6 ± 4.0%) (all p < 0.05). CMR diastolic parameters were independent predictors of TTE-based diastolic dysfunction after adjusting for left ventricular hypertrophy, hypertension, and coronary artery disease. Good correlations were observed between CMR PSR_E_/PSR_A_ and early-to-late diastolic annular velocity ratios (e′/a′) measured by TDI (r = 0.756 to 0.828, p < 0.001).

**Conclusions:**

3D MA sweep volumes generated by semi-automated AVJ tracking in routinely acquired CMR images yielded diastolic parameters that were effective in identifying patients with diastolic dysfunction when correlated with TTE-based variables.

## Background

Left ventricular (LV) diastolic dysfunction refers to mechanical abnormalities that impede effective volume filling during diastole. This is the main mechanism of heart failure with preserved ejection fraction (HFPEF), which accounts for up to 50% of overall cases of heart failure [1]. Despite the absence of systolic dysfunction, patients with HFPEF still experience high rates of morbidity and mortality [2]. Factors associated with diastolic dysfunction include hypertrophic, infiltrative, and dilated cardiomyopathies, advanced age, coronary artery disease (CAD), and systemic hypertension (HTN) [3]. Moreover, diastolic dysfunction often precedes systolic dysfunction, and is a sensitive indicator of early coronary and hypertensive heart disease [4]. As a result, the characterization of diastolic function, even in asymptomatic subjects, may have important implications for prognosis and treatment strategies.

Transthoracic echocardiography (TTE) is currently the standard method used in the noninvasive evaluation of diastolic function. Commonly, pulsed-wave Doppler of transmitral flow (TMF) is used, with pulmonary venous flow (PVF) and tissue Doppler imaging (TDI), to assess myocardial relaxation and filling pressures. However, TTE measurements have limitations, due to variability in sampling locations and ultrasound beam alignment [5]. In particular, TDI-derived velocities are position dependent, and may not accurately reflect global diastolic function in the presence of regional dysfunction [6]. One study demonstrated that the average of four annular site measurements is more accurate than a single site measurement [7]. While x-ray computed tomography has also been introduced as a potential way to assess diastolic function [8], this is still investigational.

Cardiovascular magnetic resonance (CMR) has emerged as a promising alternative modality for quantifying diastolic dysfunction [9]. CMR's good spatial resolution, field of view, and range of tissue contrasts allow correlation of cardiac function with morphologic and tissue characteristics. Measurements analogous to TTE measurements, such as TMF, PVF, and myocardial tissue velocities, can be performed with velocity phase-contrast CMR, which has demonstrated excellent agreement with Doppler [10,11]. Similar agreement with TTE has also been reported for CMR derived LV volume-time curves [12,13]. In addition, myocardial tagging [14] methods have had promising results. However, despite recent advances, CMR-based methods for diastology remain in their infancy. The existing methods require prolonged imaging acquisition and post-processing times, preventing routine use of CMR in assessing diastolic function [15].

Mitral annular (MA) dynamics has been previously studied with various methods [16-18]; however, the relationship between CMR MA motion and diastolic function remains largely unexplored. In a prior study, a 2D manual tracking technique in assessing CMR MA motion had been reported [19]. In this paper, we aimed to expand on this prior technique and identify patients with diastolic dysfunction by using 3D MA sweep volumes calculated from routinely acquired long-axis cine-CMR images, comparing the results to TTE.

## Methods

### Patient population and study design

The research protocol was approved by the local Institutional Review Board. Study subjects were identified from retrospectively reviewed clinical data of all consecutive patients who underwent both clinically indicated TTE and CMR at New York University Langone Medical Center between June 2011 and December 2013. Inclusion criteria were: (1) cine-CMR performed within 2 months of TTE, (2) TTE included assessment of diastolic function, and (3) normal LV systolic function on both CMR and TTE (EF > 50%). Patients were excluded for: (1) incomplete CMR images for MA reconstruction, (2) poor CMR image quality due to arrhythmias (atrial fibrillation, ventricular ectopy), or (3) sinus tachycardia that rendered interpretation of the cardiac phases difficult [9]. Clinical data at the time of imaging were collected, including symptoms, CAD history, presence of structural heart disease on CMR, and cardiovascular risk factors such as HTN, hyperlipidemia, diabetes, and tobacco use. A positive history of CAD was defined as: reports of myocardial infarction, evidence of disease on diagnostic tests, or previous revascularization.

Study subjects were divided into normal diastolic function and diastolic dysfunction groups, using TDI velocities as reference. According to echocardiography guidelines [20,21], diastolic dysfunction was defined as septal e′ MA peak velocity < 8 cm/s, lateral e′ MA peak velocity < 10 cm/s, or maximal LA volume index ≥ 34 mL/m^2^. Abnormal LA dilatation was defined as LA volume index ≥ 29 mL/m^2^ [22].

### Imaging protocol

#### *Echocardiography*

TTE was performed using conventional equipment: Philips iE33 (Phillips Medical Systems, MA, USA), General Electric Vivid 7 (General Electric Medical Systems, WI, USA), or Siemens SC2000 (Siemens Medical Solutions, CA, USA). Standard apical and parasternal views were obtained. Function and dimensional measurements were performed according to accepted guidelines [22]. TMF velocities were recorded with pulsed-wave Doppler sampling at the mitral valve leaflet tips in the apical 4-chamber view. TDI was performed with pulsed-wave Doppler sampling at the MA junctions of the septal and lateral walls in the apical 4-chamber view.

#### CMR

Conventional cine-CMR (2D steady state free precession pulse sequence) was performed using 1.5 or 3 T MRI systems (Avanto, Tim Trio, Siemens Medical Solutions, Erlangen, Germany) with a 6-element phased array anterior receiver coil, as part of routine clinical protocol. Cine images were acquired in multiplanar short-and long-axis views with retrospective electrocardiographic gating. Cine short-axis views were used for routine evaluation of global cardiac function. Typical imaging parameters were as follows: TR/TE = 2.4/1.4 ms, temporal resolution = 37–63 ms, in-plane spatial resolution = 1.4 mm × 1.4 mm - 1.8 mm × 1.8 mm, flip angle = 51°, slice thickness = 6 mm, receiver bandwidth = 930 Hz/pixel, and 25 reconstructed phases per cardiac cycle.

### CMR image analysis

#### AVJ tracking and interpolated 3D reconstruction of mitral annulus

**Figure 1:**
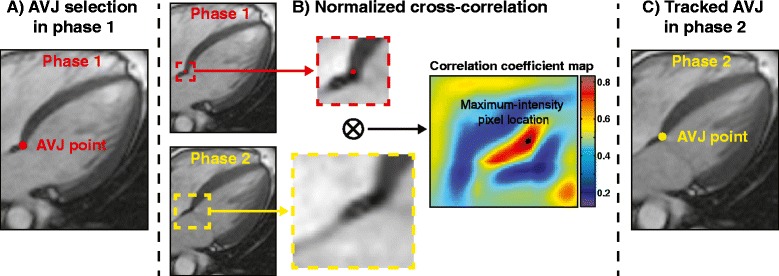
**Semi-automated AVJ tracking in cine-CMR. (A)** User selects initial atrioventricluar junction (AVJ) point (highlighted in red) in cardiac phase 1 of long-axis cine-CMR series. **(B)** A template (red square) centered on the AVJ point in phase 1 undergoes normalized cross-correlation (NCC) with a larger region of interest (ROI) (yellow square) in phase 2. Calculation of NCC produces a correlation coefficient map, in which the maximum-intensity pixel location corresponds to new AVJ location in phase 2. **(C)** The new AVJ location is used as the template for the next iteration, and this process is repeated for all remaining phases in the image series.

The atrioventricular junction (AVJ), the septal and lateral junction between the left atrium and ventricle, was tracked in two-, three-, and four-chamber long-axis CMR views over the cardiac cycle [19]. This was performed semi-automatically in lab-written MATLAB (MathWorks Inc., MA, USA) using normalized cross-correlation (NCC), a well-known feature-tracking algorithm [23,24] that has been used in MA location tracking in CMR [18]. Briefly, the user initially selects an AVJ point in the image of cardiac phase 1 (Figure 1A). This point becomes the center of a square template (red square) that undergoes NCC with a larger ROI (yellow square) in the image of phase 2 (Figure 1B), producing a correlation coefficient map (empirically, the use of a 20-pixel square in the initial phase and a 40-pixel square in the subsequent phase produced good tracking results that were not sensitive to the precise number of pixels used in this study).

In this calculated correlation map, the location of the maximum-intensity pixel indicates the best corresponding location of the initial template feature in the new ROI, which is thus taken as the AVJ location in phase 2 (Figure 1C). This new point becomes the center of a new template that then undergoes similar calculation of the NCC with the corresponding ROI in the next phase; the process is similarly automatically repeated for the subsequent phases, in order to track the AVJ locations throughout the cardiac cycle. The software also allows for optional interactive user correction of the AVJ location, should there be tracking errors (e.g., due to blurring artifacts). After user correction for a specific phase, if needed, automated AVJ tracking with the NCC method is then performed again for the subsequent phases in the image series, using the corrected location as the starting point.

**Figure 2:**
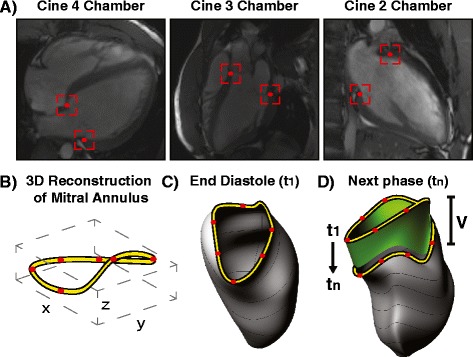
**Interpolated 3D reconstruction of mitral annulus. (A)** AVJ points were tracked in two-, three-, and four-chamber cine-CMR views to create six distinct spatial points (highlighted in red) sampled in the mitral annulus (MA) per cardiac phase. **(B)** 3D spline interpolation was applied to the 3D space locations of these points to create a 3D reconstruction of the mitral annulus (MA). **(C, D)** A 3D MA sweep volume (V, highlighted in green) was generated for each cardiac phase relative to the MA area at t_1_ (end diastole), by summing incremental volumes calculated from the MA area at that phase and the distance the MA traversed from the previous phase.

For each cardiac phase, two AVJ locations were tracked in each of the two-, three-, and four-chamber long-axis CMR views (Figure 2A), creating six independent spatial 3D coordinates within the AVJ that were tracked over the cardiac cycle. Note that 2D image coordinates were transformed into the corresponding 3D space coordinates, using spatial information about the image acquisition locations from DICOM headers. A 3D spline curve was then used to interpolate these 6 distinct 3D spatial coordinates sampled within the MA at each cardiac cycle phase, in order to create a reconstruction of the MA in 3D space (Figure 2B), using solid-modeling software Rhinoceros (McNeel, WA, USA). This is analogous to methods that have been used to reconstruct the 3D shape of the MA from ultrasound images [25]. 3D spline curves have been similarly used to reconstruct the 3D MA structure in prior CMR studies [18,26]. A 3D MA incremental sweep volume (V_n_) was then generated for each cardiac phase t_n_, using the MA areas at t_n-1_ and t_n_, and the 3D distance (positive or negative) through which the MA traversed (Figure 2C, D); the net sweep volume at a given cardiac phase was derived from the sum of the incremental volumes starting from end-diastole.

#### Diastolic function

**Figure 3:**
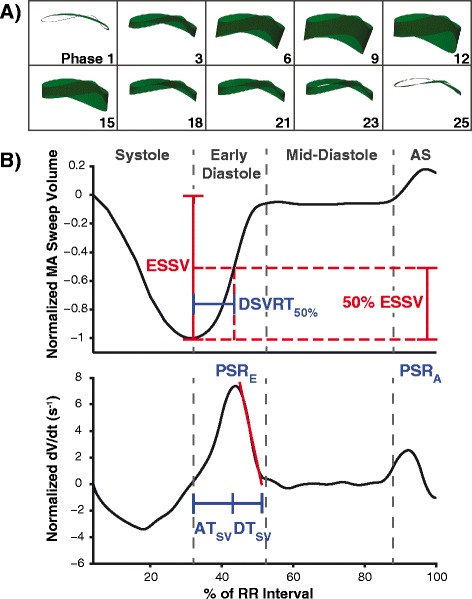
**Mitral annulus sweep volume curve and associated diastolic parameters. (A)** Representative 3D models of MA sweep volumes at different phases of the cardiac cycle. **(B)** Representative MA sweep volume (upper) and sweep rate (lower) profiles from a subject with normal diastolic function. Both curves were normalized to end-systolic sweep volume (ESSV). Cardiac intervals were identified based on the transitions in slope of the sweep volume curve, distinguishing systole, early diastole, mid-diastole, and atrial systole (AS). 50% diastolic sweep volume recovery time (DSVRT_50_) was measured as the time required in diastole for the MA to recover 50% of ESSV, and was adjusted for RR interval. Normalized peak sweep rates in early diastole (PSR_E_) and atrial systole (PSR_A_) are shown in the sweep rate curve. Sweep volume acceleration time (AT_SV_) was measured from onset of early diastole to the time of PSR_E_, and sweep volume deceleration time (DT_SV_) was measured by linear extrapolation of PSR_E_ to baseline.

The resultant MA sweep volume curve was manually divided into its corresponding cardiac cycle intervals according to transitions in its slope: systole, early diastole (ED), mid-diastole (MD), and atrial systole (AS) (Figure 3). In addition, its first derivative was calculated to characterize the MA sweep volume rate in each interval. For comparisons between different heart sizes, which may confound the absolute amounts of myocardial relaxation, the sweep volumes (and rates) were normalized to the corresponding end systolic sweep volume (ESSV). To quantify measures related to LV myocardial relaxation, the following CMR diastolic parameters were derived from the curves: (1) peak sweep rates (PSR) in ED and AS; (2) average sweep rates in ED, MD, AS; (3) percentage sweep volume recovery in ED and AS, defined by the proportion of ESSV that the MA had recovered during the given interval; and (4) the ratio of peak sweep rate in ED to peak sweep rate in AS (PSR_E_/PSR_A_). Note that PSR_E_/PSR_A_ is analogous to the e′/a′ ratio used in TDI.

Three time-interval parameters were selected to characterize diastolic function from the sweep volumes: (1) acceleration time (AT_SV_), measured from ED onset to the time of PSR_E_; (2) deceleration time (DT_SV_), measured by linear extrapolation of PSR_E_ to baseline; and (3) 50% diastolic sweep volume recovery (DSVRT_50_), defined as the time required in diastole for the MA to recover 50% of its end systolic sweep volume (adjusted for RR interval); this index had been introduced in a prior study to describe CMR LV volume filling [12].

### Statistical analysis

All statistical analyses were performed using SPSS version 20.0 (SPSS Inc., Chicago, Illinois). Continuous variables were presented as mean ± SD, whereas categorical data were presented as frequencies with percentages. Differences between groups were evaluated using either the Student t-test for normally distributed data, or the Mann–Whitney U-test for asymmetrically distributed data; Shapiro-Wilk test was used to assess normality of distributions. Fisher's exact test was used to compare differences in categorical data. In addition, the association between CMR- and TTE-based parameters was assessed using the Spearman's rank correlation test. For classification performance, receiver operating characteristic (ROC) analyses were used to evaluate the ability of CMR parameters to identify patients with TTE-based diastolic dysfunction. Optimal cutoff values for these parameters were obtained, along with their associated sensitivity and specificity. Multivariate logistic regression was used to determine the predictive ability of CMR parameters with respect to TTE-based outcomes, after controlling for age, HTN, left ventricular hypertrophy (LVH), and CAD status. A two-tailed value of p < 0.05 was considered statistically significant.

Intraobserver and interobserver variability were evaluated for 10 randomly selected subjects using intraclass correlation coefficients (ICC), Bland-Altman analyses, and Pearson's r correlation coefficients. For interobserver variability assessment, analyses were performed by two independent CMR readers who were blinded to TTE and each other's results. For intraobserver variability assessment, analyses were performed by one user who re-analyzed the same 10 subjects after a 3 week interval.

## Results

### Population characteristics

A total of 124 consecutive subjects met the selection criteria and were included in the study. The time difference between the dates of CMR and TTE was 16 ± 16 days. Based on TTE findings, subjects were classified as either having normal diastolic function (n = 62) or diastolic dysfunction (n = 62).

Table 1 details the baseline characteristics of the study population. Compared to the normal diastolic function group, those with TTE-based diastolic dysfunction were older in age (61 ± 14 vs. 40 ± 14 years, p < 0.001) and were more likely to be male (p = 0.046). Clinically, the diastolic dysfunction group had increased frequencies of HTN (61% vs. 26%, p < 0.001), hyperlipidemia (56% vs. 27%, p = 0.002), and CAD (35% vs. 11%, p = 0.003). In terms of symptoms, patients with diastolic dysfunction were more likely to complain of dyspnea (31% vs. 15%, p = 0.052). A higher proportion of subjects in the diastolic dysfunction group, compared to those in the normal diastolic function group, had CMR evidence of structural heart disease (76% vs. 32%, p < 0.001); in particular, the prevalence of LVH was significantly higher in the diastolic dysfunction group (39% vs. 10%, p < 0.001). These clinical findings were consistent with prior reports on the prevalence of diastolic dysfunction [27]. Morphologically, TTE-derived LV mass index and LA volume index were both significantly higher in the diastolic dysfunction group.

**Table 1:** **Population characteristics**

	**Overall**	**Normal diastolic function‡ (n = 62)**	**Diastolic dysfunction‡ (n = 62)**	**P**
**Clinical**				
Age, years	51 ± 17	40 ± 14	61 ± 14	<0.001*
Male, n (%)	76	31 (49%)	45 (65%)	0.046*
Heart Rate, bpm	71 ± 12	72 ± 12	71 ± 12	0.628
**Cardiovascular risk factors**				
Hypertension, n (%)	54	16 (26%)	38 (61%)	<0.001*
Hyperlipidemia, n (%)	52	17 (27%)	35 (56%)	0.002*
Diabetes mellitus, n (%)	9	3 (5%)	6 (10%)	0.491
Tobacco use, n (%)	45	19 (31%)	26 (42%)	0.262
Coronary artery disease, n (%)	29	7 (11%)	22 (35%)	0.003*
**Cardiovascular symptoms**				
Angina, n (%)	32	18 (29%)	14 (23%)	0.539
Dyspnea, n (%)	28	9 (15%)	19 (31%)	**0.052†**
Palpitation/Arrhythmia, n (%)	29	15 (24%)	14 (23%)	1.000
Syncope/Pre-syncope, n (%)	20	10 (16%)	10 (16%)	1.000
Asymptomatic, n (%)	35	20 (32%)	15 (24%)	0.425
**Structural heart disease on CMR**				
Normal, n (%)	57	42 (68%)	15 (24%)	<0.001*
Ischemic heart disease, n (%)	10	3 (5%)	7 (11%)	0.323
Congenital heart disease, n (%)	4	3 (5%)	1 (2%)	0.619
Infiltrative disease, n (%)	5	1 (2%)	4 (6%)	0.365
LV hypertrophy, n (%)	30	6 (10%)	24 (39%)	<0.001*
Dilated cardiomyopathy, n (%)	2	0 (0%)	2 (3%)	0.496
Inflammatory cardiomyopathy, n (%)	9	5 (8%)	4 (6%)	1.000
**Function measures by TTE**				
Interventricular septal wall thickness, mm	1.11 ± 0.39	0.96 ± 0.32	1.25 ± 0.41	<0.001*
Inferolateral wall thickness, mm	0.98 ± 0.18	0.9 ± 0.15	1.06 ± 0.17	<0.001*
LV end-diastolic diameter, mm	4.52 ± 0.6	4.69 ± 0.54	4.35 ± 0.61	0.001*
LV end-systolic diameter, mm	2.86 ± 0.52	2.94 ± 0.49	2.78 ± 0.55	0.102
LV mass index, g/m^2^	87 ± 27	80 ± 21	95 ± 31	0.010*
LV ejection fraction,%	64 ± 6	62 ± 6	65 ± 7	**0.059†**
LA volume index, ml/m^2^	25 ± 9	22 ± 7	27 ± 10	0.001*
LA dilatation, n (%)	30	6 (10%)	24 (39%)	<0.001*
**Function measures by CMR**				
LV end-diastolic volume, mL	152 ± 39	157 ± 40	147 ± 39	0.211
LV end-systolic volume, mL	59 ± 20	62 ± 19	57 ± 20	0.234
Stroke volume, mL	93 ± 24	95 ± 24	90 ± 24	0.242
Cardiac output, L/min	7.4 ± 8.7	8.5 ± 12	6.2 ± 2	0.241
Cardiac index, L/min/m^2^	3.3 ± 0.9	3.4 ± 0.9	3.2 ± 0.9	0.101
LV ejection fraction,%	61 ± 6	61 ± 5	62 ± 7	0.720

Data expressed as mean ± SD or frequencies (percentages). LV = Left ventricular; LA = Left atrial.

*P < 0.05, †P < 0.10, ‡Patient classification based on echocardiography reference.

### AVJ tracking and MA reconstruction

Our CMR method successfully generated MA sweep volume curves for all subjects in the study. The approximate processing time for each case was less than 3 minutes. The processing included the initial user delineation of AVJ points, review and possible correction of semi-automated tracking results, MA reconstruction in 3D, and the identification of cardiac intervals within the sweep volume curve. Manual corrections of AVJ locations were primarily necessary in images with blurring artifacts affecting the regions of interest. Approximately 30% of cine image series required at least one manual correction. Cases that did not require manual correction were analyzed in under 1 minute.

**Figure 4:**
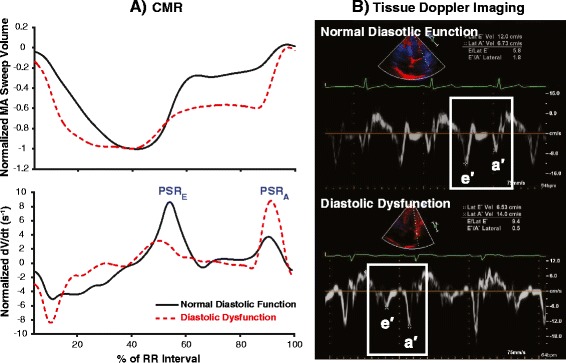
**Graphical comparisons between normal diastolic function and diastolic dysfunction in diastole. (A)** Representative MA sweep volume and sweep rate profiles of patients with TTE-based normal diastolic function (solid line) and diastolic dysfunction (dashed line) as generated by the proposed CMR method. Apparent differences in relative diastolic indices are seen in normalized peak sweep rates during early diastole (PSR_E_) and atrial systole (PSR_A_). **(B)** Corresponding tissue Doppler imaging, showing qualitatively similar relative lateral MA velocity profiles between the same two patients. Note that the patient with diastolic dysfunction had a decreased early diastolic annular velocity (e′) but an increased atrial systole velocity (a′), resulting in e′/a′ reversal that is typical of diastolic dysfunction.

Figure 4A illustrates representative patients, comparing the normalized MA sweep volume curves and their derivatives between subjects with normal diastolic function and diastolic dysfunction. These curves show that the MA of a diastolic dysfunction subject requires more time to recover from its end systolic volume state, as quantified by the parameter DSVRT_50_. Furthermore, the derivative curve provides a visual representation of the normalized MA sweep rate at each cardiac interval. In normal diastolic function, there was typically a higher MA sweep rate during early diastole that was accompanied by a relatively lower sweep rate during atrial systole; this was reversed in diastolic dysfunction. In these cases, peak MA sweep rates (PSR_E_ and PSR_A_) on CMR were in qualitative relative agreement with the echocardiographically-derived early (e′) and late (a′) TDI annular velocity profiles (Figure 4B). In addition, the utility of DT_SV_ was demonstrated in the sweep volume derivative curve, where the diastolic dysfunction subjects showed a relative flattening of the deceleration component in early diastole.

### LV diastolic function

Table 2 describes the CMR and TTE diastolic parameters for the two groups. Compared to patients with TTE-based normal diastolic function, those with TTE-based diastolic dysfunction had slower normalized peak and average MA sweep rates in early diastole (5.3 ± 1.4 s^−1^ vs. 7.7 ± 1.7 s^−1^ and 2.5 ± 0.6 s^−1^ vs. 4.2 ± 1.0 s^−1^, respectively, both p < 0.001). The diastolic dysfunction group also had a lower PSR_E_/PSR_A_ ratio (0.9 ± 0.4 vs. 1.8 ± 0.7, p < 0.001), due to their relatively higher peak sweep rate in atrial systole. During early diastole, the MA of hearts with diastolic dysfunction recovered a smaller proportion of their end systolic sweep volume (54 ± 12% vs. 69 ± 10%, p < 0.001), and their MA sweep volume constituted a smaller percentage of their stroke volume (7.6 ± 2.2% vs. 11.2 ± 2.9%, p < 0.001). Diastolic dysfunction was also differentiated by its longer DT_SV_ (144 ± 55 ms vs. 96 ± 37 ms, p < 0.001) and longer DSVRT_50_ (25 ± 11% vs. 16 ± 4.0%, p < 0.001).

**Table 2:** **Diastolic parameters**

	**Normal diastolic function‡ (n = 62)**	**Diastolic dysfunction‡ (n = 62)**	**P**
**CMR mitral annulus sweep volume**			
Normalized peak sweep rate, early diastole (PSR_E_), s^−1^	7.72 ± 1.7	5.25 ± 1.38	<0.001*
Peak sweep rate, early diastole, cm^3^/s	117 ± 43	65 ± 28	<0.001*
Normalized peak sweep rate, atrial systole (PSR_A_), s^−1^	4.67 ± 1.38	6.56 ± 1.99	<0.001*
Peak sweep rate, atrial systole, cm^3^/s	70 ± 24	80 ± 29	0.031*
PSR_E_/PSR_A_ ratio	1.82 ± 0.69	0.9 ± 0.44	<0.001*
Percentage sweep volume recovery, early diastole	69.09 ± 10.02	54.29 ± 12.37	<0.001*
Absolute sweep volume, early diastole, cm^3^	10.64 ± 4.1	6.83 ± 2.66	<0.001*
Absolute sweep volume/stroke volume, early diastole, %	11.17 ± 2.89	7.6 ± 2.21	<0.001*
Percentage sweep volume recovery, atrial systole	33.78 ± 9.92	49.1 ± 13.76	<0.001*
Absolute sweep volume, atrial systole, cm^3^	5.03 ± 1.72	6.01 ± 2.35	0.014*
Absolute sweep volume/stroke volume, atrial systole, %	5.41 ± 1.78	6.81 ± 2.24	0.014*
Normalized average sweep rate, early diastole, s^−1^	4.2 ± 1.02	2.53 ± 0.63	<0.001*
Normalized average sweep rate, mid-diastole, s^−1^	0.36 ± 0.71	0.87 ± 1.34	0.019*
Normalized average sweep rate, atrial systole, s^−1^	2.67 ± 0.94	3.88 ± 1.26	<0.001*
Sweep volume deceleration time (DT_SV_), ms	96.25 ± 36.56	144.19 ± 54.89	<0.001*
Sweep volume acceleration time (AT_SV_), ms	102.48 ± 35.03	114.2 ± 41.09	**0.066†**
50% Diastolic sweep volume recovery time (DSVRT_50_), %	15.58 ± 4	24.97 ± 11.03	<0.001*
**Echocardiography**			
E, cm/s	84.47 ± 22.22	75.81 ± 25.08	0.020*
A, cm/s	59.45 ± 17.75	69.9 ± 22.7	0.005*
E/A	1.51 ± 0.51	1.22 ± 0.67	<0.001*
Deceleration time, ms	219 ± 48	230 ± 47	0.200
e′ septal, cm/s	10.14 ± 2.22	6.05 ± 1.4	<0.001*
a′ septal, cm/s	8.25 ± 1.89	8.98 ± 2.44	**0.064†**
e′/a′ septal	1.3 ± 0.41	0.75 ± 0.34	<0.001*
e′ lateral, cm/s	14.59 ± 3.95	8.1 ± 2.56	<0.001*
a′ lateral, cm/s	9.51 ± 2.95	10.4 ± 3.75	0.141
e′/a′ lateral	1.7 ± 0.71	0.89 ± 0.44	<0.001*
E/e′ (average)	7.1 ± 2.02	11.47 ± 6.26	<0.001*

Data expressed as mean ± SD.

Peak and average sweep rates were normalized to end-systolic sweep volume. Percentage sweep volume recovery measured the proportion of recovery in reference to end-systolic sweep volume. 50% diastolic sweep volume recovery time was normalized to RR interval.

E = peak early mitral inflow velocity; A = peak late mitral inflow velocity; e′ = early diastolic mitral annular velocity; a′ = late diastolic mitral annular velocity; MA = mitral annulus.

*P < 0.05, †P < 0.10, ‡Patient classification based on echocardiography reference.

**Figure 5:**
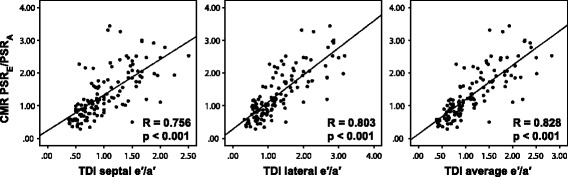
**Correlation between CMR and TDI parameters.** Spearman's rank test showed good positive correlations between CMR normalized peak volume sweep rates and TDI MA velocities in early-to-late diastolic ratios. CMR PSR_E_/PSR_A_ correlated better with TDI lateral e′/a′ than it did with TDI septal e′/a′. However, PSR_E_/PSR_A_ correlated best with the average e′/a′ of lateral and septal locations.

Figure 5 illustrates the correlations of MA velocities between CMR and TDI measured at different locations. Although there was a strong positive correlation between CMR PSR_E_/PSR_A_ and echo septal e′/a′ (r = 0.756, p < 0.001), the correlation between PSR_E_/PSR_A_ and lateral e′/a′ was even stronger (r = 0.803, p < 0.001). The strongest correlation, however, was seen between PSR_E_/PSR_A_ and the average e′/a′ of septal and lateral positions (r = 0.828, p < 0.001).

### Classification performance of CMR parameters

Table 3 summarizes the ROC analysis of CMR diastolic parameters in the identification of TTE-based diastolic dysfunction. Based on areas under the curve (AUC), PSR_E_ and PSR_E_/PSR_A_ ratio demonstrated excellent classification power (both AUC = 0.88), as did DT_SV_ (AUC = 0.83) and DSVRT_50_ (AUC = 0.79). The optimal cutoff value for PSR_E_ was 6.7 s^−1^, with a sensitivity of 89% and specificity of 74%; the optimal cutoff for PSR_E_/PSR_A_ was 1.17, with a sensitivity of 84% and specificity of 81%. In comparison, DT_SV_ and DSVRT_50_ exhibited slightly lower accuracies, with optimal cutoffs at 101 ms and 19.45%, respectively. A recovery threshold of 50% of end-systolic sweep volume was chosen for the parameter DSVRT_50_ because this generated the best classification performance compared to other recovery thresholds (thresholds of 40%, 60%, 70%, and 80% produced AUC's ranging from 0.60 to 0.75).

**Table 3:** **Classification performance of CMR diastolic parameters**

	**AUC**	**Test + Criterion**	**Sensitivity**	**Specificity**	**PPV**	**NPV**	**Accuracy**
**PSR**_**E**_	0.88	< 6.65	89%	74%	77%	87%	81%
**Percentage sweep volume recovery, early diastole**	0.82	< 57.90	65%	87%	83%	71%	76%
**Normalized average sweep rate, early diastole**	0.93	< 3.12	85%	87%	87%	86%	86%
**PSR**_**E**_ **/PSR**_**A**_	0.88	< 1.17	84%	81%	81%	83%	82%
**DT**_**SV**_	0.83	> 100.89	82%	74%	76%	81%	78%
**DSVRT**_**50**_	0.79	> 19.45	63%	84%	80%	69%	73%

Classification performance results based on receiver operating characteristic (ROC) analysis. Threshold based on value yielding the greatest classification accuracy.

PSR_E_ = normalized peak sweep rate in early diastole; PSR_A_ = normalized peak sweep rate in atrial systole; DT_SV_ = sweep volume deceleration time; DSVRT_50_ = 50% diastolic sweep volume recovery time; AUC = area under the curve; PPV = positive predictive value; NPV = negative predictive value.

### CMR prediction of diastolic dysfunction

Multivariate logistic regression analysis demonstrated that PSR_E_, PSR_E_/PSR_A_, and DSVRT_50_ were significant independent predictors of TTE-based diastolic dysfunction after controlling for age, HTN, LVH, and CAD status (Table 4). DT_SV_ was near significant as an independent predictor with an odds ratio of 1.12 per 10 ms increment (p = 0.077). In addition, PSR_E_/PSR_A_ had an odds ratio of 0.86 per 0.1 increment (p = 0.016), whereas PSR_E_ and DSVRT_50_ had odds ratios of 0.54 (p = 0.007) and 1.14 (p = 0.013), respectively.

**Table 4:** **Multivariate analysis to predict TTE-based diastolic dysfunction**

	**Odds Ratio**	**95% CI**	**Coefficient (B)**	**P**
**Model for PSR**_**E**_				
Age	1.07	1.02-1.12	0.06	0.009*
HTN	1.06	0.34-3.32	0.06	0.919
LVH	9.42	2.15-41.2	2.24	0.003*
CAD	1.22	0.35-4.21	0.20	0.758
PSR_E_	0.54	0.34-0.85	−0.62	0.007*
**Model for PSR**_**E**_ **/PSR**_**A**_				
Age	1.07	1.02-1.13	0.07	0.005*
HTN	0.96	0.31-2.94	−0.04	0.944
LVH	9.24	2.00-42.6	2.22	0.004*
CAD	0.93	0.27-3.24	−0.07	0.913
PSR_E_/PSR_A_ (per 0.1 increment)	0.86	0.76-0.97	−0.15	0.016*
**Model for DT**_**SV**_				
Age	1.10	1.05-1.15	0.09	<0.001*
HTN	1.05	0.35-3.18	0.05	0.927
LVH	10.45	2.44-44.8	2.35	0.002*
CAD	1.18	0.35-4.02	0.17	0.791
DT_SV_ (per 10 ms increment)	1.12	0.99-1.28	0.12	**0.077†**
**Model for DSVRT**_**50**_				
Age	1.08	1.04-1.13	0.08	<0.001*
HTN	1.23	0.39-3.87	0.20	0.726
LVH	12.18	3.12-47.6	2.50	<0.001*
CAD	1.04	0.29-3.77	0.04	0.950
DSVRT_50_	1.14	1.03-1.26	0.13	0.013*

Results based on multivariate logistic regression analysis.

CI = confidence interval.

*P < 0.05, †P < 0.10.

**Figure 6:**
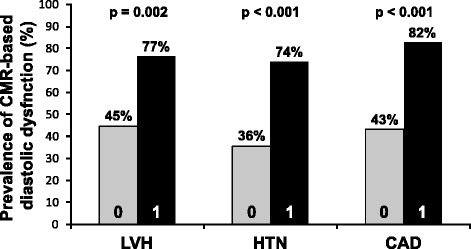
**Prevalence of CMR-based diastolic dysfunction based on clinical status.** A predictive model was constructed with multivariate logistic regression, using PSR_E_, PSR_E_/PSR_A_, DT_SV_, and DSVRT_50_ as covariates to diagnose CMR-based diastolic dysfunction in patients with (1) and without (0) left ventricular hypertrophy (LVH), hypertension (HTN) and coronary artery disease (CAD). A higher prevalence of CMR-based diastolic dysfunction was observed in these clinical subgroups.

CMR diastolic parameters were used as covariates to construct predictive models with logistic regression. A model including both PSR_E_ and PSR_E_/PSR_A_ showed an improved AUC of 0.895 in predicting TTE-based diastolic dysfunction. When adding in DT_SV_ and DSVRT_50_, the AUC improved slightly to 0.896. The latter model with all four parameters was used to demonstrate the prevalence of CMR-based diastolic dysfunction in a number of important clinical conditions in our sample population (Figure 6). As shown, the prevalence of CMR-based diastolic dysfunction was almost twice as great in patients with LVH compared to patients without LVH (p = 0.002). Similar results were observed in patients with HTN and CAD. In addition, the same predictive model showed that LA dilatation was seen in 24 of 65 subjects (37%) with CMR-based diastolic dysfunction and 6 of 59 (10%) subjects with CMR-based normal diastolic function (p < 0.001). Based on ROC analyses, LA volume index demonstrated only moderate classification powers in predicting both CMR-based and TTE-based diastolic dysfunction (AUC = 0.64, AUC = 0.67, respectively).

### Reproducibility

Table 5 shows the intraobserver and interoberserver variabilities using ICC, the Bland-Altman test, and Pearson's r correlation. CMR diastolic parameters demonstrated good consistency in terms of correlations (ICC ranged from 0.88 to 0.97) with minimal degree of biases for both intraobserver and interobserver measurements.

**Table 5:** **Reproducibility of CMR sweep volume indices**

	**Intraobserver (cases = 10)**			**Interobserver (cases = 10)**		
	**ICC**	**Bias**	**R**	**ICC**	**Bias**	**R**
	**(95% CI)**	**(Limits of agreement)**		**(95% CI)**	**(Limits of agreement)**	
**PSR**_**E**_	0.954	−0.356	0.92	0.964	−0.138	0.93
	(0.823 to 0.988)	(−1.98 to 1.268)		(0.858 to 0.991)	(−1.578 to 1.302)	
**PSR**_**E**_ **/PSR**_**A**_	0.974	−0.126	0.99	0.972	−0.021	0.94
	(0.838 to 0.994)	(−0.450 to 0.197)		(0.887 to 0.993)	(−0.501 to 0.461)	
**DT**_**SV**_	0.931	3.427	0.87	0.928	−0.143	0.87
	(0.728 to 0.983)	(−34.167 to 41.021)		(0.704 to 0.982)	(−31.609 to 31.324)	
**DSVRT**_**50**_	0.886	2.33	0.92	0.880	1.441	0.85
	(0.568 to 0.971)	(−7.171 to 11.831)		(0.527 to 0.970)	(−11.371 to 14.251)	

ICC = Intraclass correlation coefficient with 95% confidence interval (CI); bias and limits of agreement generated by Bland–Altman analysis; R correlation coefficient determined by Pearson's r test.

## Discussion

This study demonstrates that CMR diastolic parameters derived from 3D MA sweep volumes were reproducible and could accurately differentiate between patients with normal diastolic function and diastolic dysfunction, as established by TDI. CMR-based measurements of peak sweep rates were also strongly correlated with analogous TDI velocity indices. Diastolic assessments using CMR 3D MA sweep volumes were validated in a diverse population of 124 subjects with normal systolic function measures, which revealed an increased prevalence of CMR based diastolic dysfunction in patients with HTN, CAD, and LVH. This suggests that MA sweep volume may contribute to the evaluation of LV diastolic function, potentially providing additional prognostic information and guidance that could be useful in management before frank heart failure occurs [28].

Our method addresses several limitations encountered by current CMR methods of assessing diastolic function. Notably, routine use of phase-contrast imaging and tagging have been hindered by the need for additional imaging pulse-sequences and associated acquisition time. In addition, manual calculation of LV volumetric curves requires extensive post-processing effort and has associated subjectivity, while automatic measurements of these curves require proprietary and complex image-processing algorithms [13]. Our novel CMR method may provide a more practical option, since it assesses diastolic function by using conventional cine-CMR long-axis images that are already routinely acquired in clinical CMR examinations, without the need for additional imaging. This also enables users to analyze pre-existing cases.

To facilitate the measurement of MA sweep volumes, AVJ tracking was performed semi-automatically, using a simple NCC feature-tracking algorithm. NCC is an image-processing technique that has been used for many applications in motion tracking. NCC provides a means to assess the degree of similarity ("correlation") between two images, as a function of pixel position [23]. Assuming that the immediate surroundings of a given point in an initial image provide the features of a template, the algorithm "slides" and centers this template at each pixel within a neighborhood of this point in a subsequent image, and a correlation coefficient is calculated between the template and the subsequent image for each such pixel position. This process produces a map of the correlation between the point (and its surroundings) in the initial image and the points within that corresponding neighborhood in the subsequent image; the location of the maximum correlation represents the likely location of the initial point (and its surrounding template) within the subsequent image. In this study, NCC enabled semi-automated AVJ tracking that reduced the long post-processing time previously required with manual AVJ tracking [19]. In addition, the use of reproducibility analyses here showed that the use of NCC limited variability in calculated MA sweep volume measurements between users.

Our results showed that patients with TTE-based diastolic dysfunction had lower normalized peak and average MA sweep rates in early diastole, resulting in the reversal of PSR_E_/PSR_A_ ratios. Likewise, prior echocardiographic studies have reported the analogous phenomenon of TDI e′/a′ MA velocity reversal in diastolic dysfunction [21,29]. The present study also showed a relative increase in peak MA sweep rate in atrial systole in these patients. This can potentially be explained by the effect of LA dilatation associated with diastolic dysfunction, which may have led to compensatory enhancement of LA contraction, due to activation of the Frank-Starling mechanism [21]. Based on our data, LA dilatation was significantly associated with both CMR-based and TTE-based diastolic dysfunction compared to their respective controls. In addition, ROC analysis showed that the combined use of PSR_E_ and PSR_E_/PSR_A_ improved performance in identifying patients with TTE-based diastolic dysfunction. The ability to characterize atrial systole with our methods is an advantage compared to techniques such as MR tagging, which cannot reliably produce late diastolic strain rates because of fading signal intensities due to T1 relaxation [30].

MA sweep mechanics were reported here in terms of "percentage sweep volume recovery". This index showed that patients with normal diastolic function recovered nearly 70% of the end-systolic sweep volume by the end of early diastole, or 10.6 mL in absolute volume, but patients with diastolic dysfunction only recovered 54%, or 6.8 mL. These findings were in rough overall agreement with a prior study that reported an average MA excursion volume of 6 mL in nine healthy subjects [17]. However, this prior study involved subjects with a considerably lower average stroke volume (52 mL vs. 93 mL), which may explain the moderate difference in results. Overall, the CMR parameters discussed here were all consistent in demonstrating blunted early-diastolic MA kinetics in patients with abnormal TDI velocities.

We also investigated the time intervals of the MA sweep volume. The results showed that patients with TTE-based diastolic dysfunction had significantly longer DT_SV_ (144 ± 55 ms) compared to the normal diastolic function group (96 ± 37 ms). AT_SV_ was slightly longer in the diastolic dysfunction group, but the difference was not significant. Both findings were consistent with a published TDI study that examined acceleration and deceleration times pertaining to MA velocities (normal DT = 84 ms, diastolic dysfunction DT = 156 to 168 ms) [31].

DSVRT_50_ was an additional CMR diastolic function parameter, which measured the time needed for the MA to recover 50% of its end systolic sweep volume, adjusted for R-R interval. On average, longer DSVRT_50_ times were observed in patients with TTE-based diastolic dysfunction. This parameter was analogous to an index used in a prior study named "diastolic volume recovery" [12], which accounted for both heart rate and volume status when assessing diastolic dysfunction in CMR LV volumetric filling. Lastly, it is important to note that PSR_E_, PSR_E_/PSR_A_, and DSVRT_50_ each independently predicted TTE-based diastolic dysfunction after controlling for age, LVH, HTN, and CAD.

Clinically, the study demonstrated that patients with HTN, LVH, and CAD had increased prevalence of CMR-based diastolic dysfunction. Among the LVH subgroup, a large percentage (20/23 patients) was also diagnosed with hypertrophic cardiomyopathy. Similar results regarding these clinical subgroups have been reported in the past [27]. In general, HTN, LVH, and CAD are all well-known causes of diastolic dysfunction, and often co-exist in patients with cardiovascular disease. These conditions have been implicated in the disruption of myocyte calcium reuptake that governs the active phase of relaxation, as well as mechanisms that increase collagen synthesis and the ventricular stiffness that governs the passive phase of relaxation [32,33]. Because previous echocardiography studies have shown that diastolic dysfunction often develops before any clinically apparent changes of cardiac function in predisposed individuals [34], such patients undergoing routine CMR may benefit from additional assessment of their diastolic function using the proposed method. In particular, the CMR-derived diastolic function variables may contribute additional value to the conventional CMR characterization of tissue morphology and contrast agent delayed enhancement.

The MA shape was reconstructed in 3D with six imaged AVJ points, derived from three standard LV long-axis planes situated approximately 60 degrees apart in space. By accounting for MA 3D motion in its entirety, in both transverse and longitudinal directions, the proposed method was hypothesized to be a better representation of global diastolic function than TDI, which only measures longitudinal motion. Typically in TDI, longitudinal velocities are measured regionally at the septal and lateral sides of the MA. However, prior studies have reported significant regional variability in LV myocardial velocities [35]. Specifically, early TDI velocities in the lateral, inferior, and posterior locations were often greater than the septal and anterior segments. As a result, varying regional wall motion abnormalities were shown to undermine the correlation between TDI velocities and invasively acquired LV end-diastolic pressure and tau [36]. In current practice, the average of TDI velocities is often used to better characterize global LV diastolic function [6]. In this study, CMR PSR_E_/PSR_A_ correlated better with the average TDI e′/a′, compared to single-site measurements, thereby supporting the hypothesis that the proposed method inherently better characterizes global LV function. It is interesting to note that CMR PSR_E_/PSR_A_ correlated better with lateral TDI e′/a′ than it did with septal e′/a′. This finding is consistent with studies showing that, among single site measurements, lateral TDI velocities have the best correlations with global LV filling pressures and invasive indices of LV compliance [37].

The CMR-derived MA sweep volume was found here to effectively characterize global diastolic function. Prior echocardiography studies have demonstrated that MA excursion volume plays an important role in LV function [17,38]. In addition, reports have long recognized the relationship between LV long-axis function and MA displacement [39]. For example, the longitudinal MA excursion in diastole envelops and effectively transfers blood from the atrium to the ventricle, separately from the flow of blood across the location of the MA. This can occur even while the blood remains relatively stationary in relation to the apex. Similarly, in atrial systole, the MA is pulled away from the apex by the atrial pectinate muscles, "over" the blood, to facilitate further ventricular filling (and also increasing the ventricular pre-stretch, thus augmenting contractility). As a result, MA excursion is a central component of diastole, along with the transmitral pressure gradient.

### Limitations

There were some limitations in our study design. First, this was a retrospective investigation and most patients did not have their imaging studies performed on the same day. Although the subjects were in stable clinical condition between studies, changes in cardiac function between studies were possible. This may explain some discrepancy in diastolic function as identified by CMR and TTE. Other potential reasons for discrepancy include differences in temporal resolution between the methods, as well as sampling location, where regional dysfunction identified by TDI may not correspond with global dysfunction as assessed by MA sweep volume [12], or vice versa. Second, the study used TTE as the standard reference for diastolic function instead of invasive hemodynamic data. However, routine use of invasive studies is not practical. Furthermore, despite various limitations of TTE [5], it remains the generally accepted non-invasive reference method for diastolic function assessment. Third, the study lacked follow-up data that might indicate the prognostic significance of the results of the proposed method.

There are also several limitations of the proposed technique. First, the three long-axis images used to reconstruct the MA were acquired at similar, but different, points in time, e.g., with potential differences in respiratory excursion. Second, the accuracy of NCC feature-tracking can be affected by image scaling, rotation, and distortion [23]; as with other feature-tracking approaches, it is dependent on there being some suitable distinctive image structure around the point being tracked. In conventional long-axis cine-CMR images, significant scaling or rotation were rarely seen in AVJ motion. However, distortion of the AVJ was relatively common during rapid motion, due to resulting blurring artifacts, which necessitated manual correction of the AVJ tracking results in a minority of cases. Lastly, when using routinely acquired long-axis images, only six points could be used to reconstruct the 3D MA. However, a prior animal study was able to effectively characterize MA dynamics by implanting only eight equally spaced radiopaque markers on the MA [38].

Future studies involving a larger cohort with assessment of the prognostic utility of the information may offer further evaluation of the potential clinical utility of CMR-derived 3D MA sweep volume as a technique for evaluation of diastolic dysfunction. A larger population could also allow stratification of the different diastolic dysfunction stages. In addition, there may be particular value in applying the technique in evaluating specific patient groups with potential ventricular dysfunction, such as athletes, patients with hypertrophic, dilated, or restrictive cardiomyopathies, or patients undergoing anthracycline therapy.

## Conclusion

3D MA sweep volumes generated by semi-automated AVJ tracking in routinely acquired CMR images demonstrated diastolic parameters that were effective in identifying patients with TTE-based diastolic dysfunction. These parameters also correlated well with TDI MA velocities, and may serve as a useful tool for characterizing patients with diastolic dysfunction.

## Abbreviations

CMR: Cardiovascular magnetic resonance
MA: Mitral annulus
TDI: Tissue Doppler imaging
TTE: Transthoracic echocardiography
TMF: Transmitral flow
AVJ: Atrioventricular junction
PSR: Peak sweep rate
DT_SV_: Deceleration time of sweep volume
AT_SV_: Acceleration time of sweep volume
DSVRT_50_: 50% diastolic sweep volume recovery time
ESSV: End systolic sweep volume
AUC: Area-under-the-curve
ROC: Receiver-operator characteristic
ICC: Intraclass correlation coefficients
